# Social Media Use and Depressive Symptoms During Early Adolescence

**DOI:** 10.1001/jamanetworkopen.2025.11704

**Published:** 2025-05-21

**Authors:** Jason M. Nagata, Christopher D. Otmar, Joan Shim, Priyadharshini Balasubramanian, Chloe M. Cheng, Elizabeth J. Li, Abubakr A. A. Al-Shoaibi, Iris Y. Shao, Kyle T. Ganson, Alexander Testa, Orsolya Kiss, Jinbo He, Fiona C. Baker

**Affiliations:** 1 Division of Adolescent and Young Adult Medicine, Department of Pediatrics, University of California, San Francisco; 2 Factor-Inwentash Faculty of Social Work, University of Toronto, Toronto, Ontario, Canada; 3 Department of Management, Policy, and Community Health, University of Texas Health Science Center at Houston; 4 Center for Health Sciences, SRI International, Menlo Park, California; 5 School of Humanities and Social Science, The Chinese University of Hong Kong, Longgang District, Shenzhen, China

## Abstract

**Importance:**

In 2023, the US Surgeon General issued the Advisory on Social Media and Youth Mental Health, identifying critical research gaps that preclude evidence-based guidance given that most studies of social media and mental health have been cross-sectional rather than longitudinal and have focused on young adults or older adolescents rather than on younger adolescents.

**Objective:**

To evaluate longitudinal associations between social media use (time spent on social media) and depressive symptoms across 4 annual waves spanning a 3-year follow-up period from late childhood to early adolescence.

**Design, Setting, and Participants:**

In this prospective cohort study using data from the Adolescent Brain Cognitive Development Study across 21 study sites from October 2016 to October 2018, children aged 9 to 10 years at baseline were assessed across 4 waves (baseline, year 1, year 2, and year 3), with year-3 follow-up through 2022. Sample sizes varied across waves and measures due to attrition and missing data. Analyses retained all available data at each wave. Data were analyzed from January 2024 to March 2025.

**Exposures:**

Self-reported time spent on social media at baseline to 3-year follow-up.

**Main Outcomes and Measures:**

Reciprocal associations between social media use and depressive symptoms (Child Behavior Checklist) at baseline and at 1, 2, and 3 years of follow-up were assessed using longitudinal, cross-lagged structural equation panel models. Covariates included sex, race and ethnicity, household income, and parental educational level.

**Results:**

At baseline, the sample included 11 876 participants (mean [SD] age, 9.9 [0.6] years), of whom 6196 (52.2%) were male. After adjusting for stable between-person differences and covariates, within-person increases in social media use above the person-level mean were associated with elevated depressive symptoms from year 1 to year 2 (β, 0.07; 95% CI, 0.01-0.12; *P* = .01) and from year 2 to year 3 (β, 0.09; 95% CI, 0.04-0.14; *P* < .001), whereas depressive symptoms were not associated with subsequent social media use at any interval. The final random-intercept cross-lagged panel model demonstrated a good fit (comparative fit index, 0.977; Tucker-Lewis index, 0.968; root mean square error of approximation, 0.031 [90% CI, 0.029-0.033]). Between-person differences in social media use were not associated with depressive symptoms (β, −0.01; 95% CI, −0.04 to 0.02; *P* = .46) after accounting for demographic and family-level factors.

**Conclusions and Relevance:**

In this cohort study of 11 876 children and adolescents, reporting higher than person-level mean social media use in years 1 and 2 after baseline was associated with greater depressive symptoms in the subsequent year. The findings suggest that clinicians should provide anticipatory guidance regarding social media use for young adolescents and their parents.

## Key Points

**Question:**

Are there within-person associations between social media use (time) and depressive symptoms across early adolescence?

**Findings:**

In this cohort study of 11 876 children and adolescents, within-person increases in social media use during early adolescence were prospectively associated with greater depressive symptoms 1 year later, whereas depressive symptoms were not associated with later social media use.

**Meaning:**

The findings suggest that more time spent on social media during early adolescence may contribute to increased depressive symptoms over time.

## Teaser

This cohort study of children and adolescents aged 9 to 12 years evaluates person-level associations between time spent using social media and depressive symptoms over subsequent years.

## Introduction

Social media use among adolescents has risen sharply in recent years, raising concerns about its impact on mental health.^1^ In 2021, 42% of adolescents reported persistent feelings of sadness or hopelessness, an increase of 50% from 2011.^2^ Although correlations between social media use and depressive symptoms have been previously identified, the directionality of this relationship remains unclear.^3,4,5,6,7,8,9^ In 2023, the US Surgeon General issued the Advisory on Social Media and Youth Mental Health,^10^ calling for longitudinal research, as most prior studies have been cross-sectional and were therefore unable to determine temporality, directionality, or within-person changes.^3,4,5,6,7,8,9^ Disentangling whether social media use contributes to or is a reflection of preexisting distress is critical for guiding evidence-based interventions and policy decisions.

Building on these concerns, the Differential Susceptibility to Media Effects Model (DSMM)^11^ provides a guiding framework for understanding the relationship between social media use and adolescent mental health. The DSMM posits that media effects are not uniform but depend on dispositional, developmental, and sociocultural factors, which in adolescence may include heightened cognitive and emotional reactivity.^12,13^ These sensitivities make adolescence a critical period of vulnerability during which social media exposure may have lasting implications for mental health.^14^ Social media use may also play a bidirectional role; it can influence future mood states while also being shaped by preexisting depressive symptoms, potentially creating reinforcing cycles of use and distress.^15^ By applying the DSMM to examine within-person changes over time,^16^ this study aimed to identify whether there are bidirectional associations between social media use and depressive symptoms in early adolescence. Of note, the few existing longitudinal studies on social media and mental health in adolescents have reported mixed findings,^17^ and bidirectional relationships remain understudied.

To address this gap, we leveraged data from the Adolescent Brain Cognitive Development (ABCD) Study, an ongoing national prospective cohort study that tracks participants over multiple time points.^18,19^ This design enabled us to explore individual trajectories and within-person variability in the association between social media use and depressive symptoms. The design also accounted for autoregressive effects and allowed us to explore the stability of social media use and depressive symptoms over time. We hypothesized that social media use and depressive symptoms in early adolescence would exhibit bidirectional within-person associations over time.

## Methods

### Study Population

**Figure 1.**
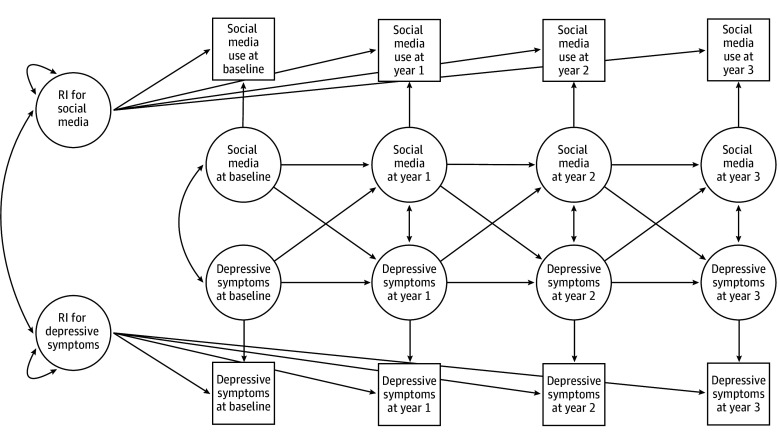
Conceptual Random-Intercept (RI) Cross-Lagged Panel Model of Social Media Use and Depressive Symptoms

In this cohort study, we conducted analyses of data from baseline to year-3 follow-up of the ABCD Study (5.1 release). The ABCD Study is the largest longitudinal study of adolescent health, brain, and cognitive development in the US. It recruited children aged 9 to 10 years from 21 sites from October 2016 October 2018 (baseline). Participants were assessed across 4 waves (baseline, year 1, year 2, and year 3), with year-3 follow-up through 2022. Specifically, the repeated assessments allowed for a clearer examination of potential directionality—whether changes in social media use preceded shifts in depressive symptoms or vice versa (Figure 1). Sample sizes varied across waves and measures due to attrition and missing data. Analyses retained all available data at each wave. The ABCD Study sample, recruitment, protocol, and measures were reported previously^20^ and are further described in the eMethods in Supplement 1. Centralized institutional review board approval for the ABCD Study was received from the University of California, San Diego; written assent was obtained from the study participants, and written consent was obtained from their parents or guardians. The current study was a secondary analysis of deidentified ABCD Study and thus did not require additional approval or assent and consent. The current study followed the Strengthening the Reporting of Observational Studies in Epidemiology (STROBE) reporting guideline for cohort studies.

### Measures

#### Social Media Use

Social media use was defined for this analysis as time spent on social media daily and was assessed using the ABCD Youth Screen Time Survey administered each year.^21^ Adolescents responded to questions about the number of hours and minutes per weekday and weekend day they spent engaging with social media. The total time spent on social media was calculated as the weighted sum: [(weekday mean × 5) + (weekend mean × 2)]/7. Weighted mean social media use was reported as a continuous variable, representing the mean daily time spent (in hours).

#### Depressive Symptoms

Depressive symptoms were assessed using the validated Child Behavior Checklist (CBCL) depressive problems score (from the *Diagnostic and Statistical Manual of Mental Disorders*–oriented scales) each year as reported by the caregiver.^22^ The raw score (as opposed to the *t* score) was chosen to capture the full distribution of symptom severity in a community-based sample and to avoid the potential loss of within-person variability over time that may occur when standardizing scores according to age- and gender-based norms. Each raw score reflects the sum of items within the depression-related subscale, with higher values indicating more severe symptoms.

#### Covariates

Several covariates were included to account for demographic and contextual factors that may be associated with social media use or depressive symptoms, including sex (female, male), race and ethnicity (ascertained by parent or caregiver report; categories were Asian, Black, Hispanic or Latino, Native American, White, or other [no defined groups, although write-ins were allowed]), household income (<$25 000, $25 000-$49 999, $50 000-$74 999, $75 000-$99 999, $100 000-$199 999, and ≥$200 000), and highest parental educational level (high school or less vs college or more). The number of adverse childhood experiences,^23^ the parental monitoring scale,^24^ family conflict (conflict subscale of the Family Environment Scale^25^), and the study site were also included as covariates (the eMethods in Supplement 1 give further details). All covariates were chosen based on prior literature suggesting their relevance to mental health and digital media behaviors.^26^

### Statistical Analysis

To examine the reciprocal associations between social media use and depressive symptoms over 4 time points, we fit several longitudinal structural equation models.^27^ We first estimated a traditional cross-lagged panel model (CLPM) and then compared it with random-intercept CLPMs (RI-CLPMs). The RI-CLPM is an extension of the traditional CLPM that explicitly separates stable between-person differences from within-person fluctuations over time.^28^ By introducing latent random intercepts, the RI-CLPM ensures that each individual’s trait-like baseline is accounted for, allowing the cross-lagged paths to focus on how temporary (state-like) deviations in one variable are associated with subsequent deviations in another. This approach is particularly valuable when examining processes that may be confounded by persistent individual differences because it isolates the time-varying relationships within persons.^16^ In doing so, the RI-CLPM clarifies whether an association is driven by people who have generally high scores on both constructs and by within-person changes that unfold across measurement occasions. It also enables the estimation of autoregressive and cross-lagged parameters that reflect carryover and spillover effects independent of any overall rank-order stability between participants.

All models were estimated using maximum likelihood with robust SEs (MLR) in the lavaan package^29^ in R, version 4.4.2 (R Project for Statistical Computing). MLR accounts for nonnormality and provides robust SEs and a scaled test statistic. Full information maximum likelihood was used to handle missing data on the outcome measures (the eMethods in Supplement 1 provide more detail on missing data). We evaluated model fit using the comparative fit index (CFI), Tucker-Lewis index (TLI), root mean square error of approximation (RMSEA), and standardized root mean square residual (SRMR). We relied on fit criteria indicating good to excellent model fit^30^ (eg, CFI ≥0.95, TLI ≥0.95, RMSEA ≤0.06, and SRMR ≤0.08). In accordance with the benchmarks of Orth et al,^31^ cross-lagged effect sizes were interpreted as small (β = 0.03), medium (β = 0.07), and large (β = 0.12). Two-sided *P* < .05 was considered significant. Analyses were performed from January 2024 to March 2025.

## Results

Sociodemographic characteristics of the analytic sample after excluding participants with missing baseline data for age or sex assigned at birth (N = 11 876) are presented in Table 1. A total of 5680 participants (47.8%) were female, 6196 (52.2%) were male, and the mean (SD) age at baseline was 9.9 (0.6) years. In all, 709 participants (6.0%) were Asian; 2392 (20.1%), Black; 2027 (17.0%), Hispanic or Latino; 410 (3.4%), Native American; 6166 (51.9%), White; and 171 (1.4%), other race and ethnicity. Table 2 shows descriptive statistics and 0-order correlations for the untransformed social media use variables and the CBCL depression raw scores across waves; descriptive indices (mean, SD, and range) suggested an overall increase in mean daily social media use from baseline to year 3 and a modest increase in mean depressive symptom scores.

**Table 1.** Sociodemographic Characteristics of ABCD Study Participants at Baseline

Characteristic	Participants, No. (%) (N = 11 876)
Sex	
Female	5680 (47.8)
Male	6196 (52.2)
Race and ethnicity^a^	
Asian	709 (6.0)
Black	2392 (20.1)
Hispanic or Latino	2027 (17.0)
Native American	410 (3.4)
White	6166 (51.9)
Other	171 (1.4)
Household income, $	
≤24 999	1633 (13.8)
25 000-49 999	1588 (13.4)
50 000-74 999	1498 (12.6)
75 000-99 999	1570 (13.2)
100 000-199 999	3311 (27.9)
≥200 000	1250 (10.5)
Parent’s highest educational level	
College or more	2039 (17.2)
High school or less	9799 (82.8)

Abbreviation: ABCD, Adolescent Brain Cognitive Development.

^a^ Reported by the parent and/or caregiver of the participant. The “other” category had no specific racial or ethnic groups defined, although write-ins were allowed.

**Table 2.** Descriptive Statistics and 0-Order Correlations of Key Variables of Social Media Use, Depressive Symptoms, and Covariates Across Baseline and 3 Follow-Up Years

Variable	*r*										Participants, No.	Mean value (SD)^a^
	1	2	3	4	5	6	7	8	9	10		
1. Social media use, baseline											11 832	0.12 (0.42)
2. Social media use, year 1	0.37										11 175	0.22 (0.60)
3. Social media use, year 2	0.28	0.44									10 370	0.68 (1.54)
4. Social media use, year 3	0.24	0.38	0.56								10 301	1.21 (2.05)
5. Depressive symptoms, baseline	0.03	0.03	0.00	0.00							11 859	1.27 (2.01)
6. Depressive symptoms, year 1	0.03	0.02	0.01	0.01	0.64						11 199	1.39 (2.19)
7. Depressive symptoms, year 2	0.03	0.04	0.05	0.04	0.55	0.62					10 895	1.50 (2.30)
8. Depressive symptoms, year 3	0.03	0.05	0.05	0.06	0.47	0.55	0.63				10 093	1.69 (2.52)
9. Adverse childhood experiences	0.08	0.06	0.06	0.07	0.20	0.18	0.16	0.16			11 865	1.71 (1.59)
10. Parental media monitoring	−0.01	0.02	0.03	0.02	−0.09	−0.10	−0.08	−0.07	−0.14		11 846	4.38 (0.52)
11. Family conflict	0.09	0.04	0.04	0.04	0.11	0.11	0.08	0.07	0.22	−0.24	11 843	2.05 (1.95)

^a^ Variables 1 through 4 are hours per day and were transformed in the final analytic model because the first 2 years were reported in ordinal categories while the last 2 were reported in continuous hours (0-24). Variables 6 through 11 are scores (details of scores are given in the Depressive Symptoms subsection of the Methods section and the eMethods in Supplement 1).

To examine the reciprocal associations among social media use and depressive symptoms over time and to concurrently partition stable, between-person differences from within-person fluctuations, we estimated 3 alternative longitudinal structural equation models. Model comparisons were conducted in a sequential manner and based on standard global fit indices.^30^ Following Chen’s^32^ recommended cutoff criteria for fit indices (eg, CFI ≥0.95, RMSEA ≤0.06, change in CFI ≤0.01, and change in RMSEA ≤0.015), we evaluated whether each model demonstrated good fit and whether differences in fit between nested models were meaningful. First, we estimated the traditional CLPM, which does not partition stable between-person variance and yielded a poor fit (CFI, 0.917; TLI, 0.807; RMSEA, 0.132 [90% CI, 0.127-0.137]; SRMR, 0.065).

Next, we evaluated the constrained RI-CLPM, which incorporates latent random intercepts to capture trait-like differences and imposes equality constraints on the cross-lagged paths across time. The constrained model provided markedly improved fit indices (CFI, 0.966; TLI, 0.957; RMSEA, 0.036 [90% CI, 0.034-0.038]; SRMR, 0.027). Compared with the traditional CLPM, the constrained RI-CLPM showed improved model fit (change in CFI, –0.049; change in RMSEA, 0.096). In addition, we estimated the unconstrained RI-CLPM, which allowed the cross-lagged parameters to vary freely across time. This model showed further improvement in fit (CFI, 0.977; TLI, 0.968; RMSEA, 0.031 [90% CI, 0.029-0.033]; SRMR, 0.022). Compared with the constrained RI-CLPM, the unconstrained version improved model fit modestly (change in CFI, 0.011; change in RMSEA, −0.005). On the basis of these comparisons and given the theoretical merit of allowing time-specific associations,^14^ we retained the unconstrained RI-CLPM as our final model (eTable 1 in Supplement 1). In the unconstrained RI-CLPM, social media use and depressive symptoms were modeled across the 4 waves (baseline, year 1, year 2, and year 3), controlling for between-person variance and the covariates listed in the Methods.

### Between-Person Association

eTable 2 in Supplement 1 displays the standardized estimates for fixed covariates of both social media use and depressive symptoms within the final model. There were no between-person associations between depressive symptoms and social media use, as evidenced by the covariance between the latent random intercepts (β, −0.01; 95% CI, −0.04 to 0.02; *P* = .46). This suggests that adolescents with consistently high (or low) social media use were not necessarily the same adolescents with consistently high (or low) depressive symptoms after accounting for demographic and familial factors and within-person estimates.

### Within-Person Associations

**Figure 2.**
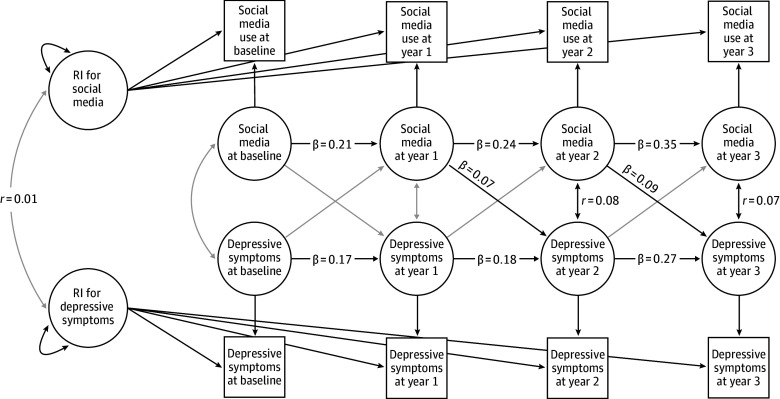
Results of Random-Intercept (RI) Cross-Lagged Panel Model of Social Media Use and Depressive Symptoms in the Adolescent Brain Cognitive Development Study Black lines represent statistically significant cross-lagged and autoregressive paths and gray lines, nonsignificant paths.

The time-varying within-person variables were modeled by estimating both autoregressive and cross-lagged paths among the residuals of the observed indicators (Figure 2 and eTable 3 in Supplement 1). Autoregressive effect sizes were significant for both constructs. Social media use exhibited strong temporal continuity, with an autoregressive coefficient (β) of 0.21 (95% CI, 0.16-0.25; *P* < .001) from baseline to year 1, 0.24 (95% CI, 0.20-0.27; *P* < .001) from year 1 to year 2, and 0.35 (95% CI, 0.32-0.38; *P* < .001) from year 2 to year 3. Depressive symptoms also showed stability, with autoregressive coefficients of 0.17 (95% CI, 0.14-0.20; *P* < .001) from baseline to year 1, 0.18 (95% CI, 0.14-0.21; *P* < .001) from year 1 to year 2, and 0.27 (95% CI, 0.24-0.31; *P* < .001) from year 2 to year 3. Within-wave residual covariance was modeled to assess contemporaneous associations. At year 3, the standardized residual covariance between social media use and depressive symptoms was 0.07 (95% CI, 0.03-0.10; *P* < .001), meaning that individuals reporting social media use higher than the person-level mean at that wave also tended to have depressive symptoms higher than the person-level mean.

The cross-lagged paths, reflecting how deviations from an individual’s expected level on one variable are associated with subsequent deviations on the other, revealed no association between depressive symptoms at baseline and social media use in year 1 (β, 0.00; 95% CI, –0.01 to 0.02; *P* = .46) or between social media use at baseline and depressive symptoms in year 1 (β, 0.03; 95% CI, –0.02 to 0.09; *P* = .19). However, significant cross-lagged associations emerged in subsequent waves. Specifically, from year 1 to year 2, social media use higher than the person-level mean in year 1 was associated with greater depressive symptoms in year 2 (β, 0.07; 95% CI, 0.01-0.12; *P* = .01; medium effect size). This pattern continued from year 2 to year 3, in which social media use higher than the person-level mean in year 2 was positively associated with greater depressive symptoms in year 3 (β, 0.09; 95% CI, 0.04-0.14; *P* < .001; medium effect size). There were no cross-lagged associations between depressive symptoms and later social media use at either interval (eg, year 1 depressive symptoms and year 2 social media use: β, 0.00 [95% CI, –0.007 to 0.01]; *P* = .65; year 2 depressive symptoms and year 3 social media use: (β, 0.00 [95% CI, –0.003 to 0.02]; *P* = .18).

## Discussion

In this cohort study of children and adolescents aged 9 to 12 years in the US, we found that there was a longitudinal association between increases in social media use and subsequent depressive symptoms at the within-person level. These findings provide initial evidence of temporal ordering and could suggest that social media use is a potential contributing factor to adolescent depressive symptoms rather than merely a correlate or consequence of such symptoms. Our findings are consistent with prior studies that have found associations between social media use and depression.^33,34,35^ In addition, a meta-analysis of 21 cross-sectional and 5 longitudinal studies found that there was a linear dose-response association between social media use and depression, further suggesting that social media use may be a risk factor for depression.^36^

Only a limited number of studies have assessed bidirectional longitudinal associations between social media use and depression in adolescents, with mixed findings.^37,38,39^ One Australian study of individuals aged 10 to 17 years used the RI-CLPM to analyze data from 2013 to 2015 and found no significant cross-lagged associations between social media use and depression.^38^ A Dutch study of data from 2016 to 2018 (mean [SD] participant age of 13.1 [0.8] years) using the RI-CLPM found a unidirectional association between problematic social media use and decreased mental health a year later but not vice versa.^39^ However, that study found no longitudinal associations between social media use intensity (eg, frequency of viewing, messaging) and mental health in either direction.^39^ In contrast, our study found that more time spent on social media, conceptually close to the Dutch study’s measure of intensity, was associated with later depressive symptoms. Potential reasons for the differing findings could include variations in the periods (adolescent social media use has increased significantly in the past 15 years), age ranges (the analytic cohort in our study was limited to individuals aged 9 to 12 years), and country (US, Australia, and the Netherlands).

These findings can be interpreted within the context of the DSMM,^11^ which posits that some adolescents may be more susceptible to negative media effects due to dispositional (eg, personality, self-esteem), developmental (eg, age), and social-contextual factors (eg, family conflict). Differential susceptibilities may also explain why some social media may be beneficial for certain individuals while detrimental to others. In later waves of our study (particularly year 3), adolescents who reported social media use higher than the person-level mean also showed depressive symptoms higher than the person-level mean in the same wave. The contemporaneous associations suggest that immediate factors (eg, negative peer social interactions, family conflict) may coincide with or amplify concurrent distress.

The present study’s findings have implications for clinical practice and health policy. When interpreting our findings within the context of the DSMM,^11^ interventions targeted at addressing developmental and social-contextual factors that may be associated with the negative effects of social media among adolescents could be considered. In particular, given that age is a likely developmental factor associated with these negative outcomes, early detection of and intervention for social media use are important. Furthermore, although our findings suggest that there is a unidirectional association between social media use and depression, with increases in social media use associated with depressive symptoms through subsequent years, prior research on adolescents, especially those with depressive symptoms, has shown that they can shift from maladaptive to more positive patterns of social media use when they become aware of its impact on their mood.^40^ Specifically, qualitative interviews with treatment-seeking adolescents with depression revealed that over time, many adjusted their social media behaviors, reducing stress-related posting, avoiding triggering content, and using social media more intentionally to connect with supportive peers.^41^ Interventions that promote mindful, purpose-driven social media use, such as encouraging adolescents to prioritize social connection,^41^ may help mitigate negative outcomes and support better mental health.

Clinicians should consider inquiring about social media use among children and adolescents, particularly those younger than the recommended age limits (the minimum age requirement for most social media platforms is 13 years), and providing anticipatory guidance as needed. Professional organizations, such as the American Academy of Pediatrics, could refine guidelines on social media use and emphasize the importance of family media plans and intentional social media use.

### Strengths and Limitations

The strengths and limitations of this study should be noted. Our study adds to knowledge in the field of adolescent health and communication science by examining longitudinal associations between social media use and depressive symptoms over 4 years, whereas most previous studies were cross-sectional. In addition, strengths include the analysis of a large, demographically diverse, contemporary sample of children and adolescents in the US. Limitations include the observational design of the study, leading to susceptibility to residual and unmeasured confounders despite adjustment for potential confounders, as well as reporting, recall, and social desirability bias.^42^

## Conclusions

In this cohort study of participants enrolled in the ABCD Study at age 9 or 10 years, higher person-level social media use in years 1 and 2 was associated with greater depressive symptoms in years 2 and 3. These findings suggest that more time spent on social media during early adolescence may contribute to increased depressive symptoms over time. Future studies could examine whether social media use is linked to heightened depressive symptoms by examining short-term shifts in cognitive (eg, negative self-talk, social comparison, or rumination) and excitative (eg, physiological arousal or stress) states, both of which are outlined as mediators in the DSMM.^11^ Given that these states may fluctuate over days, weeks, or seasons, more intensive within-person designs (eg, daily diaries, ecological momentary assessment, and passive mobile sensing via smartphone) may offer a more precise understanding of these processes compared with annual assessments. Future research should also continue to examine the prospective relationships between social media and mental health outcomes as the ABCD Study cohort ages to middle and late adolescence as well as aim to examine the mechanisms underlying these associations.

## Notes

Supplement 1. **eMethods.** Study Design, Sample, Missing Data, Model Comparison and Construction, and Fixed Covariates **eTable 1.** Model Fit Indices for Traditional, Constrained, and Unconstrained Random-Intercept Cross-Lagged Panel Models **eTable 2.** Fixed Covariates in the Unconstrained Random-Intercept Cross-Lagged Panel Model for Social Media Use and Depressive Symptoms **eTable 3.** Estimated Residual Covariances, Autoregressive Paths, and Cross-Lagged Associations Between Social Media Use and Depressive Symptoms in the Unconstrained Random-Intercept Cross-Lagged Panel Model
eReferences

Supplement 2.
Data Sharing Statement
