# Supplementary material for: Social Media Use and Depressive Symptoms During Early Adolescence
Source: JAMA Netw Open. 2025 May 21;8(5):e2511704. doi: 10.1001/jamanetworkopen.2025.11704 (PMC12096259; doi:10.1001/jamanetworkopen.2025.11704)
Supplement: Supplement 1. — eMethods. Study Design, Sample, Missing Data, Model Comparison and Construction, and Fixed Covariates eTable 1. Model Fit Indices for Traditional, Constrained, and Unconstrained Random-Intercept Cross-Lagged Panel Models eTable 2. Fixed Covariates in the Unconstrained Random-Intercept Cross-Lagged Panel Model for Social Media Use and Depressive Symptoms eTable 3. Estimated Residual Covariances, Autoregressive Paths, and Cross-Lagged Associations Between Social Media Use and Depressive Symptoms in the Unconstrained Random-Intercept Cross-Lagged Panel Model eReferences [file jamanetwopen-e2511704-s001.pdf]

Supplementary Online Content

Nagata JM, Otmar CD, Shim J, et al. Social media use and depressive symptoms during early adolescence. *JAMA Netw Open*. 2025;8(5):e2511704.
doi:10.1001/jamanetworkopen.2025.11704

eMethods. Study Design, Sample, Missing Data, Model Comparison and Construction, and Fixed Covariates
eTable 1. Model Fit Indices for Traditional, Constrained, and Unconstrained Random-Intercept Cross-Lagged Panel Models
eTable 2. Fixed Covariates in the Unconstrained Random-Intercept Cross-Lagged Panel Model for Social Media Use and Depressive Symptoms
eTable 3. Estimated Residual Covariances, Autoregressive Paths, and Cross-Lagged Associations Between Social Media Use and Depressive Symptoms in the Unconstrained Random-Intercept Cross-Lagged Panel Model
eReferences

This supplementary material has been provided by the authors to give readers additional information about their work.

© 2025 Nagata JM et al. *JAMA Network Open*.

eMethods. Study Design, Sample, Missing Data, Model Comparison and Construction, and Fixed Covariates

Study Design

The Adolescent Brain Cognitive Development (ABCD) Study is a nationwide, multi-site cohort study aimed at examining brain development and adolescent health among 11,875 children recruited from 21 diverse locations across the United States. To reduce selection bias, participants were primarily identified and enrolled through schools, ensuring consideration of factors such as sex assigned at birth, race/ethnicity, socioeconomic background, and urbanicity.[1] Specifically, the study utilized a probability-based approach to select schools within these 21 recruitment regions, strategically distributing them across the country's four primary geographic areas (Northeast, South, Midwest, and West) to enhance demographic and socioeconomic diversity.

To guide recruitment, the ABCD Study leveraged annual datasets maintained by the National Center for Education Statistics, which provided detailed sociodemographic information on students attending both public and private institutions within the designated catchment areas. Schools were chosen using a stratified probability sampling method, helping to mitigate systematic recruitment biases at the institutional level. As a result, while the study's sampling approach is grounded in epidemiological principles, it does not fully represent the entire U.S. population due to the voluntary nature of research participation and potential self-selection biases. Additionally, the study's weighting methodology does not account for variations in participation rates at individual schools or response rates among students.[2] Further descriptions of the ABCD Study's recruitment process and design have been detailed in prior publications.[1]

Measures: Covariates

Adverse childhood experiences (ACEs)

We calculated the ACEs score defined as the number of ACEs using responses from adolescents and parents in the ABCD Study baseline survey, which assessed nine out of ten ACEs adapted from the original CDC-Kaiser surveys.[3] These included household divorce or separation, household substance use, household criminal justice involvement, household mental illness, household violence, physical abuse, sexual abuse, emotional neglect, and physical neglect. The ACEs score was determined by summing the "yes" responses from either the adolescents or caregivers for any of the nine subtypes of ACEs at baseline, based on prior research. A "yes" response indicated that the participant had ever experienced that event in their lifetime.[4]

Parental monitoring scale

Parental monitoring was assessed using validated measures of children's perceptions of parental monitoring.[5–7] Youth responded on a Likert scale ranging from never (1) to almost always (5). The final score was calculated as the average of five questions, ranging from 1 to 5, with higher scores indicating greater parental monitoring behaviors.[7,8]

Family conflict score

Family conflict was assessed using youth responses to the Conflict subset of the Family Environment Scale.[6,7,9] The score was calculated as the average of nine questions evaluating conflict between family members, including parents and children. The scale ranged from 0 to 9, with higher scores indicating greater family conflict. The family conflict score demonstrated good test-retest reliability (ICC = 0.49) and acceptable internal consistency ($\alpha = 0.68$).[7,8]

Sample

The sample included 5,680 females (47.8%) and 6,196 males (52.2%). Participants identified their race/ethnicity as White (n = 6,166, 51.9%), Latino (n = 2,027, 17.0%), Black (n = 2,392, 20.1%), Asian (n = 709, 6.0%), Native American (n = 410, 3.4%), and Other (n = 171, 1.4%), with 1 participant missing data on race/ethnicity. Highest parental education level was high school or less for 2,039 participants (17.2%) and college or more for 9,799 (82.8%), with 38 participants (0.3%) missing data. Household income was reported as <$25,000 for 1,633 participants (13.8%), $25,000-$49,999 for 1,588 (13.4%), $50,000-$74,999 for 1,498 (12.6%), $75,000-$99,999 for 1,570 (13.2%), $100,000-$199,999 for 3,311 (27.9%), and $200,000 or more for 1,250 (10.5%), with 1,026 participants (8.6%) missing data.

Missing Data

As part of our preliminary analyses, we first evaluated whether the study data met the criteria for missing completely at random (MCAR) using a Hawkins test[10] via the MissMech package[11]. Results indicated that the assumption of MCAR was rejected at the $p < 0.05$ level, suggesting that the missingness in our dataset is unlikely to be completely

© 2025 Nagata JM et al. *JAMA Network Open*.

random. Next, to investigate whether the data could be considered missing at random (MAR)[12], we examined correlations between a missingness indicator (i.e., whether participants completed all waves of data collection) and both key study variables and demographic characteristics. These analyses revealed that missingness was significantly, albeit modestly, associated with baseline social media use ($r = -0.059$), baseline depressive symptoms ($r = -0.035$), as well as parental education ($r = 0.162$) and household income ($r = 0.155$). A logistic regression further confirmed that participants with higher baseline social media use and depressive symptoms, and those with lower education or income, were slightly less likely to have complete data. Although these findings indicate that missingness depends in part on observed covariates, they are broadly consistent with a MAR mechanism.[12] Because our primary models include these variables—and because we employ FIML[13]—the impact of missing data on our hypothesis tests is expected to be minimal. Descriptive statistics showed a highly skewed distribution (skew > 3.5 in some waves), with many participants reporting zero time on social media. To mitigate skewness and ensure more normal-like distributions, we log-transformed the screen-time variables so that zero values would remain valid.[14] Following log-transformation, each time point's log-transformed variable was standardized into a z-score so that estimates could be interpreted in standard deviation units. All primary analyses (e.g., cross-lagged panel models) used the z-scored, log-transformed social media variables and the raw CBCL depression variables at each wave. This approach allowed us to test within-person changes and between-person differences in social media use and depressive symptoms in a manner that was robust to non-normality and retained clinically relevant variation in symptomatology.

Covariates

For social media use, significant predictors included sex assigned at birth ($\beta = -0.22$, $p < 0.001$), race/ethnicity ($\beta = 0.05$, $p < 0.001$), parental education ($\beta = -0.10$, $p < 0.001$), household income ($\beta = -0.06$, $p < 0.001$), adverse childhood experiences (ACEs) ($\beta = 0.02$, $p < 0.001$), parental media monitoring ($\beta = 0.05$, $p < 0.001$), and family conflict ($\beta = 0.01$, $p < 0.001$). Study site was not a significant predictor of social media use ($p = 0.087$). Regarding depressive symptoms, significant predictors included sex assigned at birth ($\beta = 0.12$, $p < 0.001$), parental education ($\beta = 0.26$, $p < 0.001$), household income ($\beta = -0.03$, $p = 0.014$), ACEs ($\beta = 0.20$, $p < 0.001$), parental media monitoring ($\beta = 0.14$, $p < 0.001$), and family conflict ($\beta = 0.07$, $p < 0.001$). Race/ethnicity ($p = 0.956$) and study site ($p = 0.124$) were not significant predictors of depressive symptoms.

© 2025 Nagata JM et al. *JAMA Network Open*.

eTable 1. Model Fit Indices for Traditional, Constrained, and Unconstrained Random-Intercept Cross-Lagged Panel Models

Model	CFI	TLI	RMSEA (90% CI)	SRMR	ΔCFI	ΔRMSEA
Traditional CLPM	0.917	0.807	0.132 (0.127–0.137)	0.065	–	–
Constrained RI-CLPM	0.966	0.957	0.036 (0.034–0.038)	0.027	-0.049	0.096
Unconstrained RI-CLPM	0.977	0.968	0.031 (0.029–0.033)	0.022	0.011	-0.005

Note. CLPM = Cross-Lagged Panel Model; RI-CLPM = Random-Intercept Cross-Lagged Panel Model; CFI = Comparative Fit Index; TLI = Tucker-Lewis Index; RMSEA = Root Mean Square Error of Approximation (90% Confidence Interval); SRMR = Standardized Root Mean Square Residual; ΔCFI = Change in Comparative Fit Index; ΔRMSEA = Change in Root Mean Square Error of Approximation.

© 2025 Nagata JM et al. *JAMA Network Open*.

eTable 2. Fixed Covariates in the Unconstrained Random-Intercept Cross-Lagged Panel Model for Social Media Use and Depressive Symptoms

Fixed Covariates	β	SE	p	95% CI	β	SE	p	95% CI
	Social Media Use (RI)				Depressive Symptoms (RI)			
Sex Assigned Birth	-0.22	0.01	<.001	[-0.24, -0.12]**	0.12	0.03	<.001	[0.06, 0.19]**
Study Site	0.00	0.00	0.09	[-0.00, 0.00]	0.00	0.00	0.12	[-0.01, 0.00]
Race/Ethnicity	0.05	0.01	<.001	[0.04, 0.06]**	0.00	0.01	0.96	[-0.03, 0.03]
Parental Education	-0.10	0.02	<.001	[-0.14, -0.06]**	0.26	0.06	<.001	[0.15, 0.38]**
Household Income	-0.06	0.01	<.001	[-0.07, -0.05]**	-0.03	0.01	0.01	[-0.06, -0.01]**
Adverse Childhood Experiences	0.02	0.00	<.001	[0.008, 0.02]**	0.20	0.01	<.001	[0.17, 0.22]**
Parental Media Monitoring	0.05	0.01	<.001	[0.04, 0.06]**	0.14	0.02	<.001	[0.11, 0.17]**
Family Conflict	0.01	0.00	<.001	[0.01, 0.02]**	0.07	0.01	<.001	[0.05, 0.08]**

© 2025 Nagata JM et al. *JAMA Network Open*.

eTable 3. Estimated Residual Covariances, Autoregressive Paths, and Cross-Lagged Associations Between Social Media Use and Depressive Symptoms in the Unconstrained Random-Intercept Cross-Lagged Panel Model

Parameter A	↔/→	Parameter B	Estimate (r or β)	SE	p	95% CI
Between-Person Association (Random Intercepts)						
Social Media Use (RI)	↔	Depressive Symptoms (RI)	-0.01	0.02	0.46	[-0.04, 0.02]
Within-Person Residual Covariances						
Social Media Use (B)	↔	Depressive Symptoms (B)	0.03	0.02	0.09	[-0.004, 0.06]
Social Media Use (Y1)	↔	Depressive Symptoms (Y1)	0.01	0.02	0.53	[-0.02, 0.04]
Social Media Use (Y2)	↔	Depressive Symptoms (Y2)	0.08	0.02	<.001	[0.04, 0.11]**
Social Media Use (Y3)	↔	Depressive Symptoms (Y3)	0.07	0.02	<.001	[0.03, 0.10]**
Within-Person Autoregressive Paths						
Social Media Use (B)	→	Social Media Use (Y1)	0.21	0.02	<.001	[0.16, 0.25]**
Social Media Use (Y1)	→	Social Media Use (Y2)	0.24	0.02	<.001	[0.20, 0.27]**
Social Media Use (Y2)	→	Social Media Use (Y3)	0.35	0.02	<.001	[0.32, 0.38]**
Depressive Symptoms (B)	→	Depressive Symptoms (Y1)	0.17	0.02	<.001	[0.14, 0.20]**
Depressive Symptoms (Y1)	→	Depressive Symptoms (Y2)	0.18	0.02	<.001	[0.14, 0.21]**
Depressive Symptoms (Y2)	→	Depressive Symptoms (Y3)	0.27	0.02	<.001	[0.24, 0.31]**
Within-Person Cross-Lagged Associations						
Depressive Symptoms (B)	→	Social Media Use (Y1)	0.00	0.01	0.46	[-0.007, 0.02]
Social Media Use (B)	→	Depressive Symptoms (Y1)	0.03	0.03	0.19	[-0.02, 0.09]
Depressive Symptoms (Y1)	→	Social Media Use (Y2)	0.00	0.01	0.65	[-0.01, 0.01]
Social Media Use (Y1)	→	Depressive Symptoms (Y2)	0.07	0.03	0.01	[0.01, 0.12]**
Depressive Symptoms (Y2)	→	Social Media Use (Y3)	0.01	0.00	0.18	[-0.003, 0.02]
Social Media Use (Y2)	→	Depressive Symptoms (Y3)	0.09	0.03	<.001	[0.04, 0.140]**

© 2025 Nagata JM et al. *JAMA Network Open*.

eReferences.

1. Garavan H, Bartsch H, Conway K, et al. Recruiting the ABCD sample: design considerations and procedures. *Dev Cogn Neurosci*. 2018;32:16-22. doi:10.1016/j.dcn.2018.04.004

2. Compton WM, Dowling GJ, Garavan H. Ensuring the Best Use of Data: The Adolescent Brain Cognitive Development Study. *JAMA Pediatr*. 2019;173(9):809-810. doi:10.1001/jamapediatrics.2019.2081

3. Felitti VJ, Anda RF, Nordenberg D, et al. Relationship of Childhood Abuse and Household Dysfunction to Many of the Leading Causes of Death in Adults. *American Journal of Preventive Medicine*. 1998;14(4):245-258. doi:10.1016/S0749-3797(98)00017-8

4. Raney JH, Testa A, Jackson DB, Ganson KT, Nagata JM. Associations Between Adverse Childhood Experiences, Adolescent Screen Time and Physical Activity During the COVID-19 Pandemic. *Academic Pediatrics*. 2022;22(8):1294-1299. doi:10.1016/j.acap.2022.07.007

5. Karoly HC, Callahan T, Schmiege SJ, Feldstein Ewing SW. Evaluating the Hispanic Paradox in the Context of Adolescent Risky Sexual Behavior: The Role of Parent Monitoring. *J Pediatr Psychol*. 2016;41(4):429-440. doi:10.1093/jpepsy/jsv039

6. Zucker RA, Gonzalez R, Feldstein Ewing SW, et al. Assessment of culture and environment in the Adolescent Brain and Cognitive Development Study: Rationale, description of measures, and early data. *Developmental Cognitive Neuroscience*. 2018;32:107-120. doi:10.1016/j.dcn.2018.03.004

7. Gonzalez R, Thompson EL, Sanchez M, et al. An update on the assessment of culture and environment in the ABCD Study®: Emerging literature and protocol updates over three measurement waves. *Developmental Cognitive Neuroscience*. 2021;52:101021. doi:10.1016/j.dcn.2021.101021

8. Taber KS. The Use of Cronbach's Alpha When Developing and Reporting Research Instruments in Science Education. *Res Sci Educ*. 2018;48(6):1273-1296. doi:10.1007/s11165-016-9602-2

9. Moos RH, Moos BS. A Typology of Family Social Environments. *Family Process*. 1976;15(4):357-371. doi:10.1111/j.1545-5300.1976.00357.x

10. Jamshidian M, Jalal S. Tests of homoscedasticity, normality, and missing completely at random for incomplete multivariate data. *Psychometrika*. 2010;75(4):649-674. doi:10.1007/s11336-010-9175-3

11. Jamshidian M, Jalal S, Jansen C. Missmech: An R package for testing homoscedasticity, multivariate normality, and missing completely at random (MCAR). *Journal of Statistical Software*. 2014;56(6):1-31. doi:10.18637/jss.v056.i06

12. Heitjan DF, Basu S. Distinguishing "missing at random" and "missing completely at random." *The American Statistician*. 1996;50(3):207-213. doi:10.2307/2684656

13. Enders CK, Bandalos DL. The relative performance of full information maximum likelihood estimation for missing data in structural equation models. *Structural Equation Modeling: A Multidisciplinary Journal*. 2001;8(3):430-457. doi:10.1207/S15328007SEM0803_5

14. Tabachnick B, Fidell L, Ullman J. Using multivariate statistics. In: Vol 6. Pearson; 2013:497-516.

© 2025 Nagata JM et al. *JAMA Network Open*.
