# Supplementary material for: Social Media Use and Depressive Symptoms During Early Adolescence
Source: JAMA Netw Open. 2025 May 21;8(5):e2511704. doi: 10.1001/jamanetworkopen.2025.11704 (PMC12096259; doi:10.1001/jamanetworkopen.2025.11704)
Supplement: Supplement 2. — Data Sharing Statement [file jamanetwopen-e2511704-s002.pdf]

Data Sharing Statement

Nagata. Social Media Use and Depressive Symptoms During Early Adolescence. *JAMA Netw Open*. Published May 21, 2025. doi:10.1001/jamanetworkopen.2025.11704

Data
Data available: Yes
Data types: Other (please specify)
Additional Information: Data used in the preparation of this article were obtained from the ABCD Study (https://abcdstudy.org), held in the NIMH Data Archive (NDA). Investigators can apply for data access through the NDA (https://nda.nih.gov/).
How to access data: Investigators can apply for data access through the NDA (https://nda.nih.gov/).
When available: With publication

Supporting Documents
Document types: Other (please specify)
Additional Information: Data used in the preparation of this article were obtained from the ABCD Study (https://abcdstudy.org), held in the NIMH Data Archive (NDA). Investigators can apply for data access through the NDA (https://nda.nih.gov/).
How to access documents: Investigators can apply for data access through the NDA (https://nda.nih.gov/).
When available: With publication

Additional Information
Who can access the data: Researchers whose proposed use of the data has been approved
Types of analyses: For a specified purpose
Mechanisms of data availability: With a signed data access agreement
